# Botanical Description of British Plants

**Published:** 1808-08

**Authors:** 


### *Botanical Description of British Plants.*

[ Continued from our last, Vol. xix. pp. 538—544.]

11. POLYPODIUM. *P. vulgare.*

*Ang.* Common Polypody.

*Gen. Desc.* Capsules disposed in distinct circular dots on the under surface of the leaf.

*Spec. Desc.* *Leaves* wing cleft; *lobes* united at the base, oblong, somewhat serrated, blunt. *Root* scaly. *Fruct.* yellowish brown, in rows, parallel to the rib of the lobes. *Old walls, shady places, at the roots of trees, v. common.* BL. June, Oct.

*Use.* The leaves have a weak ungrateful smell; and a nauseous sweet taste, leaving a kind of roughness and slight acrimony in the mouth.—*Lewis.* The root is sweetish; by long boiling it becomes bitter; when fresh it is a gentle purgative; an infusion of 6 drs. of it in half a pint of boiling water may be taken at twice.—*Withering.* The root was recommended by the ancients as a cathartic to purge away bile and melancholy, in the quantity of 2 drs. to a dose. The moderns have almost neglected it; those who still retain its use, recommend an aqueous extract in obstructions of the viscera, or, for a pectoral, a kind of Ptisan made of it.—*Lightfoot.* The root of the P. quercinum, or that which grows on the oak, has been most esteemed for medicinal use, though no just reason can be assigned for the preference. By the ancients it was employed as a purgative, and thought to be peculiarly useful in expelling bile and pituitous humours; therefore much used in maniacal melancholical disorders: but to act as a cathartic, the root must be exhibited in its recent state and in a large dose. It has also been recommended, and from its sensible qualities, probably with more advantage, as a pectoral or demulcent; thus joined with liquorice, its good effects have been experienced in coughs and asthmatic affections. It is now rarely used in this country; and tho' some French authors have cited instances of its success in mania, they have not been able to restore its ancient reputation in that calamitous disorder. *See Mem. de l'Ac. des Sciences,* 1751.—*Woodville.*

12. POLYPODIUM. *P. filix-mas.—Filix non ramosa. F. mas vulgare.*

*Ang.* Male Polypody. Male Fern.

*Gen. Desc.* *As above.*

*Spec.*

*Spec. Desc.* *Leaves* almost doubly winged. *Leafits* strap-spear-shaped; *lobes* blunt, finely serrated; *stem* and *mid-ribs* chaffy: 1 ½ to 4 feet high. *Fruct.* 3 to 8 on each lobe, in 2 rows near to its base and distant from its edges; none at the end.—*Woods, heaths, stony places.* Bl. June, Oct.

*Use.* The root of the male fern has lately been greatly celebrated for its effects upon the tape worm, or tænia lata; this vermifuge power of fern-root seems to have been known to the ancients. (*See Diosc. Theoph. Galen, Pliny, &c.*) and is since commended by Hoffman and other practical writers. However, though its virtues are thus recorded, the use of this root was very generally neglected, till in the hands of Mad. Noufer, a surgeon's widow in Switzerland, it acquired great celebrity, being employed by her as a secret specific for the cure of the tape worm: the secret was thought of so much importance as, after being examined by a deputation of the principal physicians of Paris, to be purchased by the King of France, and published by his order. The method of cure was as follows; after the patient has been prepared by an emollient clyster, and a lubricating supper, (of panada with butter and salt,) he is directed to take in the morning, while in bed, a dose of 2 or 3 drs. of the powdered root. (The dose for infants is one drachm.) The powder must be washed down with a draught of water; if this dose be thrown up, it must be repeated: and two hours after, the patient being kept fasting, a strong cathartic, composed of calomel and scammony is to be given, proportioned to the strength of the patient. If this does not operate in due time, it is to be followed by a dose of purging salts, and if the worm be not expelled in a few hours, this process is to be repeated at proper intervals. Of the success of this, or a similar mode of treatment, in cases of tænia, there can be no doubt, as many proofs of it, in this country, afford sufficient testimony, (*see Dr. Simmons's "Account of the Tænia;"* but whether the fern root or the strong cathartic be the principal agent in the destruction of the worm, may admit of a question; and the latter opinion seems to be more generally adopted by physicians. (*See Cullen M. M. art. Filix, Simmons pref. p. 7.*)" It appears, however, from some experiments made in Germany, that the tænia has in several instances been expelled by the repeated exhibition of the root, without the assistance of any purgative. *See Gmelin. consid. gen. filicum. 34, &c.*—*Woodville.* Dr. Withering observes, that he has frequently used this remedy, and seldom dom without the desired effect. The Siberians boil this plant in their ale, and they are fond of the flavour which it imparts to it.—*Withering.*

Cryptogamia. Musci.

13. FONTINALIS. *F. antipyretica.*

*Ang.* Great Water-moss.

*Gen. Desc.* Caps. nearly sitting, furnished with a veil, and surrounded by a tiled involucrum.—Male, bud-like, axillary; on the same plant.

*Spec. Desc.* *Caps.* lateral. *Leaves* acute, heeled, doubled together, disposed in 3 rows; 2 or 3 lines long, and half as broad, v. entire at the edge. *Shoots* 1 foot long or more, branched. The primary shoot sends out lateral and terminating ones, and these branch out again; floating on the water. *Caps.* on very short fruit stalks, enclosed in a leafy scaly involucrum. *Veil* conical. *Lid* conical, blunt, starting with a spring from the ripe capsule. *Fringe* surrounding a central point. *Seeds* green.—*Upon rocks and roots of trees, in brooks, rivulets, slow streams, and ponds.* BL. June, Sept.

*Use.* This plant is employed by the Scandinavians as a lining for the inside of their chimnies, to defend them against the fire: for, contrary to the nature of all other moss, this is hardly capable of burning.—*Withering.*

Cryptogamia. Hepaticæ.

14. MARCHANTIA. *M. polymorpha.*

*Ang.* Great Star-headed Marchantia.

*Gen. Desc.* Male Cal. salver-shaped: anthers numerous, imbedded in its dish. Fem. Cal. target-shaped, flowering on the under side: caps. opening at top. Seeds fixed to elastic fibres.

*Spec. Desc.* *Leaf* bluntly lobed. *Cal. of F. fl.* mostly 10 cleft. A yellowish substance resembling a lock of wool proceeds from the capsules, appearing to move within them while the seed is falling out. From 3 to 5 inches long, 1 broad, and irregularly lobed, dark-green, shining. *Fruit-st.* in the angles of the lobes, 1 to 3 inches high. *Caps.* greenish, dividing into 8 or 10 segments. On the upper surface, glass-shaped conical cups, on short pedicles, with a wide and scolloped margin, which enclose about 4 little bodies, finely serrated at the edges. In figure somewhat resembling an oak-leaf; surface reticulated.—*Wet places, shady or open, moist rocks.* BL. June, August.

*Use.*

*Use.* A decoction of the leaves in skimmed milk are recommended as good for the jaundice and other disorders of the liver. They have a strong aromatic smell and an acid taste.—*Lightfoot.*

15. Marchantia. *M. conica.*

*Ang.* Marchantia with warted leaves. Conic Mushroom.

*Gen. Desc.* *As above.*

*Spec. Desc.* *Leaves* forked, indented, blunt, pale green, slippery to the touch, creeping on the ground, dotted on the surface, producing new leaves from the ends of the old; in large clusters, with white tubercles. *Fem. cal.* somewhat egg-shaped, with about 5 cells underneath. *M. fl.* on the leaf, resembling warts. *Fruit-st.* 3 or 4 inches high, transparent, very tender. *Common cal.* 5 cells bursting at the base, often varying in number from some proving abortive. *Seeds* when ripe hanging out attached to threads.—*Shady brook-banks and rocks.* Bl. March, April.

*Use.* The leaves have a peculiar strong fragrant smell, and an acrid aromatic taste. They are supposed to possess the same attenuating quality as the first kind, but in a higher degree. They are recommended as antiscorbutic, and proper to thin the blood.—*Lightfoot.*

### Cryptogamia. Algæ.

16. Lichen. *L. calcareus.*

*Gen. Desc.* Male, scattered wart-like substances. Fem. smooth saucers or tubercles in which the seeds are imbedded.

*Spec. Desc.* *Crustaceous with tubercles; T.* black; *crust* clear white. Hard, stony, firmly fixed to the rocks, gritty when chewed, rather rough, cracked, set with minute white eminencies, white within, thickness of half a straw's breadth. *Tubercles* rarely found, scattered, not bordered, black within.—*Rocks.* Bl. Jan. Dec.

*Use.* When dried, powdered, and steeped in urine, it is used to dye scarlet by the Welch and by the inhabitants of the Orkneys; and the colour afforded by it, is said to be very fine. This species of L. is so peculiar to limestone rocks, that wherever that stone occurs among others, it may be distinguished at first view by this plant growing on it.—*Withering.*

17. LICHEN. *L. Parellus.*

*Gen. Desc. As above.*

*Spec. Desc. Crustaceous with saucers.* S. numerous, white, mealy, with yellowish white thick blunt borders, globular but depressed in the centre, larger and flatter with age; like crab's eyes. *Crust.* yellow white, thick, warty, white in its fracture, reddish when wet and rubbed to powder; wrinkled, granulated, stony to appearance, not gritty when chewed but rather tough. *Rocks, walls, stones, trunks of trees.* BL. Jan. Dec.

*Use.* From this species litmus is prepared: for which purpose it is gathered from the rocks in the North of England, and sent to London in casks. *Withering.*

18. LICHEN. *L. tartarens.*

*Ang.* Large yellow-saucered dyer's Lichen.

*Gen. Desc. As above.*

*Spec. Desc. Crustaceous with saucers.* S. yellow, with a white border sometimes scolloped, large, deeply concave. *Crust,* whitish, thickish, wide spreading, greatly wrinkled, reticulated underneath, growing on other decayed mosses. Substance rough, not gritty; acrid. It assumes various appearances: sometimes has a thinner and more uniform crust than usual, thickly covered with white tubercle-like excrescences, and free from shields, except in the centre, where they are so thickly crowded together, as to be confluent. Sometimes it grows on moss, the branches of which are surrounded with it exactly like the incrustations formed by springs abounding in a calcareous earth running over a bed of moss. *Rocks, large stones.* BL. Jan. Dec.

*Use.* In the Highlands of Scotland, it is much used to dye a fine claret or *pompadour* color: For this purpose, after scraping it from the rocks, and cleaning it, they steep it in urine for a quarter of a year: then they take it out, and form it into cakes, which they hang up in bags to dry: These cakes are afterwards pulverised, and the powder is used as the dye-stuff, with the addition of alum.—*Lightfoot.* It is common in Derbyshire, and incrusts most of the stones at Urswic Mere: it is gathered for the dyers by the peasants, who sell it for a penny a pound; they can collect twenty or thirty pounds a day. It gives a fine purple colour. *Withering.*

19. LICHEN. *L. candelarius.*

*Ang.* Yellow farinaceous or crusted Lichen.

*Gen. Desc. As above.*

*Spec. Desc. Crustaceous with both tubercles and saucers.* S. orange yellow, when young; yellow, when old; T. numerous merous, greenish if wet. *Crust.* yellow, powdery when young; when old, yellow, leafy at the edge; spreading wide to a hand-breadth, moderately thick. *Leaves* wrinkled, cloven, firmly fixed, lobes blunt, pulpy, with age uniting and becoming powdery. *Fructifications* when slightly concave or flat, of an orange yellow, bordered with a paler lemon yellow, the colour of the crust. When older the *fruct.* swell into the form of tubercles, the border disappears, and the crust changes to brown yellow. *Rocks, walls, trunks of trees, old boards, old pales.* Bl. Jan. Dec.

*Use.* In Sweden, in the province of Smolandia, this species of *L.* is scraped from the rocks by the inhabitants, and made use of, to mix with their tallow, for the purpose of making golden candles to be burned on festival days.—*Lightfoot.*

20. Lichen. *L. Omphalodes.*

*Ang.* Cork, Corker, or Arcell. Kenkerig *Welsh.*

*Gen Desc. As above.*

*Spec. Desc. Somewhat crustaceous, leaf-like, tiled loose. Saucers* dull purple, smooth within, grey on the outside and hairy, cracked at the edge. *Leaves* hoary, smooth, blunt, many-cleft, sprinkled with rising dots, interwoven about an inch long. Colour dull purple, shining, smooth, with numerous black fibres underneath.—*Rocks.* Bl. Jan. Dec.

*Use.* It has been used as a styptic.—It dyes wool of a brown-reddish color, or a dull but durable crimson or purple, paler but more lasting than that of Orchal: it is prepared by the country people in Iceland by steeping it in stale wine, adding a little salt to it, and making it up into balls with lime. Wool dyed with it, and then dipped in the blue vat becomes of a beautiful purple. With rotten oak it makes a good dark brown frize. Wool dyed with red-wood or sanders, and afterwards in corker, becomes of a dark reddish brown.—*Withering.*

21. Lichen. *L. pyxidatus. L. tuberculatus.*

*Ang.* Common cup-moss, or Lichen.

*Gen. Desc. As above.*

*Spec. Desc. Somewhat crustaceous, bearing cups shaped like a jelly glass. Cups* grey green, simple, somewhat scolloped at the edge. *Tubercles* brown. *Crust* at first granulated, in time of forming the leaves (which are of no certain figure) small, cut at the edge, greenish above, white underneath. *Tubes* half to one inch high, springing from the base of the leaves, thickest upwards, and expanding at the summit like a drinking glass; scolloped at the edge, hollow hollow of the upper expanded part separated by a partition from the hollow of the tubular part below; light grey colour, mealy.—*Var. with cups proliferous from the centre, and edge.* — *Woods, walls, heaths, rocks, old trees.* Bl. Jan. Dec.

*Use.* A decoction of this plant is frequently given by the common people to their children for the cure of hooping cough, but its good effects are not supported by sufficient testimonies.—*Lightfoot.*

22. Lichen. *L. rangiferinus.*

*Gen. Desc.* *As above.*

*Spec. Desc.* *Somewhat crustaceous, shrub-like.* *Tubercles* brown, small, roundish, shining, black when dry, on the terminations of the branches: *plant* hoary, hollow, very much branched like coral, about two inches high; terminating branches mostly turned downwards. *Branches* perforated in the forks. Light, brittle, hoary when dry; grey, green, or whitish, tender and soft when fresh; covered with mealy particles; neither leaf nor leafy crust. *Roots* difficult to find; it adheres slightly to the earth and to mosses, from which it readily separates. — *Heaths, woods, exposed mountains.* Bl. Jan. Dec.

*Use.* Without this plant the Laplanders could not exist; this is the food of the rein-deer, which grows fat upon it; and the rein-deer supplies every necessary of life for the contented inhabitants of that inhospitable climate.—*With.*

23. Lichen. *L. barbatus.*

*Eng.* Bearded Lichen.

*Gen. Desc.* *As above.*

*Spec. Desc.* *Somewhat crustaceous, thread-like.* *Tubercles* flesh-coloured, small, low; plant pendant, rather jointed; branches thread-shape, expanding two feet or more in length, branches like thread, greenish white, forming together a large bush or tail: from these threads stand out lateral fibres not pendant. *Saucers* few, rarely met with, small, flesh-coloured.—*On branches of trees.* Bloss. Jan. Dec.

*Use.* Its quality is astringent. When steeped for some time in water, it acquires an orange colour; and is used in Pennsylvania, according to Sillenius, to dye orange.—*Lightfoot.*

